# Safety of Retrograde Tibial-Pedal Access and Intervention in Patients with Single Remaining Non-Occluded Infra-Popliteal Runoff Artery

**DOI:** 10.3390/jcdd10110463

**Published:** 2023-11-15

**Authors:** Henry K. Siu, Emily Schultz, Sandrine LeBrun, Michael Liou, Tak W. Kwan

**Affiliations:** 1 Chinatown Cardiology, P.C., New York, NY 10013, USAkwancardio@aol.com (T.W.K.); 2 Department of Medicine, Division of Cardiology, Lenox Hill Hospital, New York, NY 10075, USA

**Keywords:** transpedal access, retrograde tibial-pedal access, alternate access, retrograde recanalization, peripheral artery disease, endovascular intervention

## Abstract

Background: The adaptation of retrograde tibial-pedal access for peripheral angiogram and intervention is limited by the lack of operator experience and concern for small distal vessel injury. This study evaluates the safety of the retrograde tibial-pedal access for peripheral angiogram and intervention in patients with two vessel infra-popliteal artery chronic total occlusions, where the access point is the sole remaining non-occluded infra-popliteal artery. Methods: A retrospective analysis of 5687 consecutive patients who underwent peripheral angiograms by retrograde tibial-pedal access via the single remaining non-occluded infra-popliteal artery was performed. Patients who had retrograde tibial-pedal access at the sole remaining infra-popliteal artery confirmed by angiography were included. Clinical and ultrasound data of the accessed infra-popliteal vessel up to 6 months were collected. Results: The cohort consisted of 314 patients (152 males; mean age 77.9 years). At 6 months, access vessel complications occurred in 15 patients (4.8%). Access vessel occlusion occurred in 9 out of 314 patients (2.9%), arteriovenous fistula in 4 (1.3%), with spontaneous resolution in 2, pseudoaneurysm requiring thrombin injection in 2 (0.6%) and non-cardiovascular death in 1 (0.3%). No uncontrolled bleeding, procedure-related hospitalizations or limb amputations occurred. Conclusions: Routine primary retrograde tibial-pedal access for lower extremity peripheral artery diagnostic angiography and intervention in patients with single infra-popliteal artery runoff can be safety performed in an outpatient setting with infrequent and manageable complications.

## 1. Introduction

The contemporary lower extremity endovascular approach frequently employs retrograde tibial-pedal access as a second-line strategy when traditional or antegrade trans-femoral access is unsuccessful. The retrograde tibial-pedal access for peripheral artery disease (PAD) angiography and intervention obviates the risk for vascular complication associated with the commonly accessed femoral artery. In comparison to a traditional contralateral femoral artery access, procedures via retrograde tibial-pedal access will not need the potentially difficult endeavors of navigating through disease or tortuous iliac arteries. In addition, the adaptation of retrograde tibial-pedal access is limited by the lack of operator experience and concern for small vessel injury, especially in cases with significant PAD in multiple infra-popliteal arteries.

Previous operators and studies have shown retrograde tibial-pedal access to be feasible and safe in superficial femoral artery (SFA) occlusive disease [1], as well as cohorts exhibiting a range of critical limb ischemia [2,3,4]. The adoption of the retrograde tibial-pedal access approach lies largely with the success in treatment of difficult lesions’ subtypes, including vessel occlusions, where a retrograde intervention is met with different and often more favorable lesion characteristics and potentially less engagement with collateral branches [5,6,7].

Data are lacking when a primary tibial-pedal access method is utilized as the primary access method for PAD therapy in a range of patients presenting with severe claudication and ischemic limb pain. This study aims to evaluate the safety of a first-line primary retrograde tibial-pedal access approach for peripheral angiogram and intervention by selecting a cohort with advanced infra-popliteal disease demonstrated by two vessel infra-popliteal artery chronic total occlusions (CTO), for which the access point is at the sole remaining non-occluded distal infra-popliteal artery.

## 2. Materials and Methods

**Figure 1:**
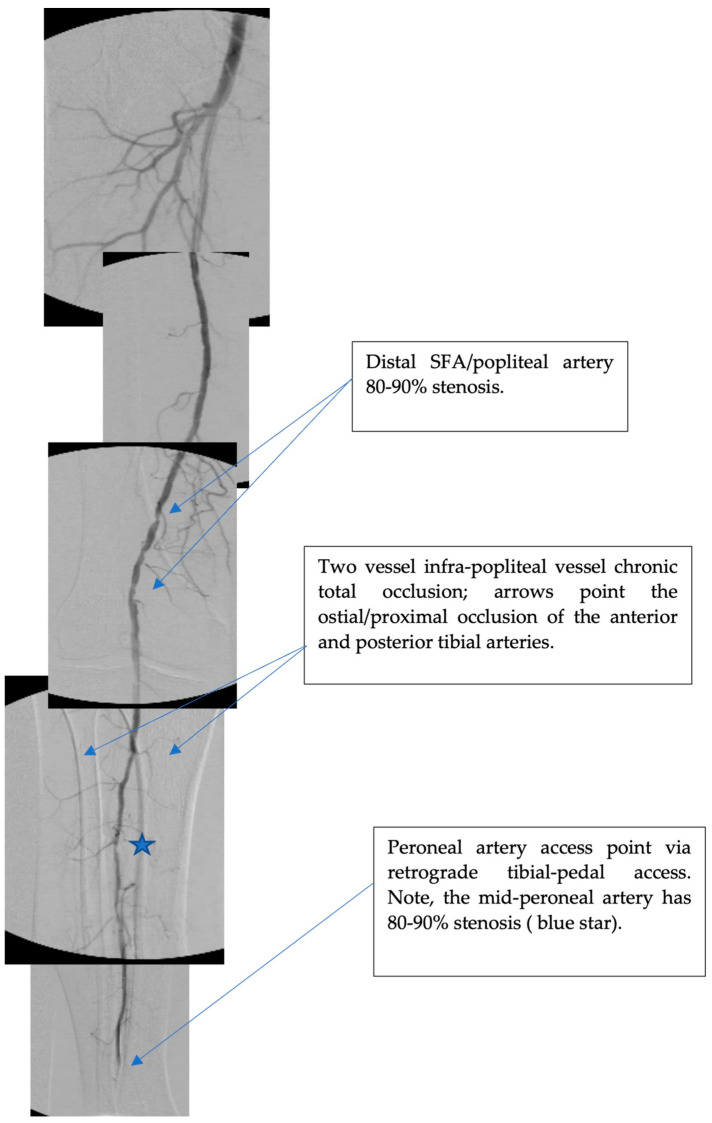
An angiogram illustrating a representative patient anatomy included in the study. This patient has 2-vessel infra-popliteal occlusions and single non-occluded infra-popliteal runoff artery which was accessed for angiogram and intervention.

We performed a retrospective cohort study from our single outpatient peripheral angioplasty center in New York City (NY, USA). Our program utilizes retrograde tibial-pedal access as the routine primary access approach for peripheral angiography and angioplasty. A retrospective analysis of 5687 consecutive patients who underwent peripheral angiograms via retrograde tibial-pedal access from June 2014 to December 2020 was completed. Patients included for analysis had 2 vessel infra-popliteal total (100%) occlusion determined by invasive angiography, for whom the sole remaining, non-occluded infra-popliteal vessel was accessed. Infra-popliteal vessels included AT, PT and peroneal arteries. Tibial-posterior trunk occlusions, which anatomically jeopardize both PT and peroneal artery flow, were also included. Figure 1 illustrates a representative patient vessel anatomy studied.

### 2.1. Patient Selection

At our center, prior to peripheral angiogram, all patients received guideline-directed conservative therapies, and the decision to proceed with invasive peripheral angiography was at the discretion of the referring cardiovascular trained physician. All procedures were thoroughly examined for case appropriateness in relation to patient symptoms, candidacy for outpatient proceeding, including status of co-morbidities, life expectancy, and severity non-invasive data including ankle-brachial index and lower extremity arterial doppler. Lower extremity CT angiography or MRA were not necessary data points prior to being offered peripheral angiography. Surgical referral and/or hospital-level care were considered for patients with large wounds and/or deemed not appropriate for outpatient-based angioplasty. Informed consent including the risk and benefits of retrograde tibial-pedal approach (our standard first-line access approach) for peripheral angiography and possible intervention was obtained from all patients. The appropriateness of all peripheral vascular interventions were guided by expert consensus and clinical practice guidelines [8,9].

### 2.2. Retrograde Tibial-Pedal Access and Hemostasis

The protocol for tibial-pedal retrograde access and hemostasis have been described in previous studies [10,11,12]. All patients were continued on their guideline-directed PAD management of anti-platelet agents (aspirin, cilostazol or oral P2Y12 inhibitors); anticoagulants were held accordingly prior to procedure.

Ultrasound guided access with a 7.5 MHz linear transducer was utilized in all patients; the peroneal artery was accessed by fluoroscopy guidance, if not optimally, with Doppler. An initial selection of 4 French (Fr) Pinnacle Precision™ or 5/6 Fr Slender™ (Terumo Medical Corporation, Somerset, NJ, USA) sheaths was placed depending on anatomy and estimated size of the access vessel. The distal segment of the infra-popliteal vessel was accessed, and in instances without an optimal access zone, a more distal pedal vessel such as the dorsalis pedis was accessed. After confirmation of arterial access, verapamil (1 mg) and nitroglycerin (200 mcg) were given through the sheath to reduce arterial spasm. Also, intravenous unfractionated heparin bolus (50 U/kg, maximum, 5000 U) was given with a target activated clotting time (ACT) of 250 s or more for intervention. In a minority of cases, at the discretion of the operator, left transradial artery access was obtained when difficulty was encountered in completing the diagnostic or intervention via the retrograde tibial-pedal access. The arterial puncture technique and pharmacotherapy is identical to the described primary access site.

Hemostasis was achieved by using a patent hemostasis technique, utilizing compression devices: TR band™ (Terumo Medical Corporation, Somerset, NJ, USA) for posterior tibial and peroneal arteries and Vasostat™ (Forge Medical, Inc., Bethlehem, PA, USA) for the anterior tibial artery. Hemostasis devices were gradually weaned off, and patients were generally discharged home 2 h after the procedure.

### 2.3. Peripheral Vascular Intervention

Angioplasty including vessel preparation, intra-vascular imaging and balloon/bailout stent sizing was at the discretion of the performing physician. Vessel preparation with orbital atherectomy was available and utilized at our center due to compatibility with a 4 or 5 French sheath size via retrograde tibial-pedal access; we utilized CSI Diamondback 1.25 Micro Crown or CSI Diamondback 1.50 Solid Crown, depending on the vessel size (Cardiovascular Systems, Inc., St Paul, MN, USA).

### 2.4. Endpoint and Analysis

Baseline patient demographics and procedural data were recorded. Clinical assessment and ultrasound data were performed at 1 week, 30 days and at 6 months post-procedure. Primary endpoints included access and procedure success rate defined by <30% angiographic residual diameter stenosis of intended treatment vessel, major adverse events, access site complications and access vessel patency rate by ultrasound. Other secondary safety endpoints including contrast volume use and fluoroscopy time were recorded.

## 3. Results

Of the 5687 consecutive patients analyzed, a total of 314 patients met the inclusion criteria. Baseline characteristics collected are outlined in Table 1. The cohort consisted of 152 males (48.4%), and 181 patients (57.6%) were of Asian descent. The mean age was 77.9 years (range 46–100 years). The access vessel distribution is outlined in Table 2, with the majority of vessels accessed being the distal AT (44.9%) and distal peroneal artery (44.3%).

Of the 314 patients included in this study, all patients had successful access via the sole remaining infra-popliteal runoff vessel. There were 306 (96.8%) patients who had an intervention performed, and all achieved pre-defined procedural success. There were 263 (83.7%) patients who had an intervention performed to the accessed infra-popliteal vessel, 267 (85%) to the popliteal artery or above vessel and 227 (72.3%) to both above- and below-knee vessels. Of the total 314 patients, 86 (27.3%) had superficial femoral artery (SFA) and/or popliteal artery total occlusions. Of the 263 patients with access vessel interventions, 238 underwent orbital atherectomy (90.5%), 240 had balloon angioplasty (91.3%) and 10 required stenting (3.8%).

As outlined in Table 3, access vessel complications occurred in 15 patients (4.8%). Of these 15 patients, all had an underlying disease with >70% stenosis and underwent intervention. Access vessel occlusion occurred in 9 out of 314 patients (2.9%). Arteriovenous (AV) fistula developed in four patients (1.3%) but with spontaneous resolution in two of these patients. Pseudoaneurysm requiring thrombin injection occurred in two patients (0.6%), and there was one non-cardiovascular death (0.3%). There were no uncontrolled bleeding events, need for blood transfusions, or limb amputations within the 6-month follow-up.

Of the 15 vessels that were reported to have complications, 12 (80%) involved the AT and 3 (20%) involved the peroneal artery. The PT artery was the access vessel in 10.2% of the patients. Of the three peroneal complications, two occurred with five French sheaths, and three of the nine AT complications occurred with five French sheaths. The average contrast use was 42 ± 12 mL and fluoroscopy time was 1231 ± 947 s.

## 4. Discussion

In this retrospective study of consecutive patients exhibiting severe infra-popliteal disease involving two occluded infra-popliteal vessels, the safety of a primary access of retrograde tibial-pedal access for peripheral angiography and intervention was demonstrated with infrequent vascular complications. This study is unique in that it selected a truly “anatomically sick” cohort with multi-level and multi-vessel runoff occlusive PAD and illustrated the safe applicability of retrograde tibial-pedal access as a routine primary access route, even in light of such diseased anatomy.

With endovascular PAD revascularization from a retrograde tibial-pedal access approach, vessel preparation and plaque modification of significant stenotic tibial disease is necessary to allow for the efficient passage of diagnostic and therapeutic devices for the above-knee segments. This may be contrary to an antegrade access approach, where diseased tibial vessels may be at the discretion of conservative therapy. In our cohort, it should be noted that the sole remaining non-occluded infra-popliteal artery that was accessed had significant obstructive lesions (>70% stenosis) in 273 (86.9%) of the total 314 patients, with an average infra-popliteal diseased length of 186.7 mm—of these, over 95% received tibial vessel angioplasty, allowing for above-knee angiography and/or intervention to optimize direct flow to the foot. Approximately ~90% of the infra-popliteal angioplasty procedures utilized orbital atherectomy, which can be safely implemented via 4 French sheaths. Treatment bias from the cohort studied and access route utilized was attributed to the high percentage of atherectomy used.

Significant popliteal artery and above-knee obstructive disease requiring endovascular treatment occurred in 85% (267 of 314) of patients. There were 27.3% of patients (86 of 314) with SFA and/or popliteal artery CTO, a severity consistent with other published literature on symptomatic PAD [13]. Furthermore, advanced techniques including Controlled Antegrade Retrograde Subintimal Tracking (CART) were also feasible, as exemplified in 12 patients with SFA CTO. These 12 cases required a second access point via the left radial artery to cross the occlusion for successful intervention. Therefore, a primary transpedal access combined with a non-femoral artery access approach can be considered in complex multi-level or multi-vessel occlusive PAD, including when more than one access route may be in the endovascular treatment plan.

Bearing in mind the outpatient setting of the analysis, this study consisted of patients presenting with a less severe spectrum of critical limb ischemia with Rutherford Class (RC) 4 in 64.5% and RC5 in 5.7% of patients; in addition, lifestyle limiting claudication RC3 was present in 28% of the patients. This study adds to the body of literature on an alternative and safe vascular access with a primary pedal-first approach, even in patients with single vessel runoff, and evades the well-studied complications of a common femoral artery access including clinically relevant bleeding and vascular complications [14,15].

Appropriate patient selection for endovascular procedures and referral for surgical revascularization are key elements to a successful outpatient endovascular program. Furthermore, adherence to guideline-directed non-procedural therapy including exercise, lifestyle and pharmacologic intervention are valuable in determining candidates for endovascular treatments and optimizing clinical outcomes [8,9]. These measures may have accounted for the very favorable outcomes including only one non-cardiovascular related death at 6 months (attributed to pneumonia), which is in contrast to other series studying a similar cohort of severe PAD and CLI [16,17,18,19].

Disruption to the access tibial pedal vessel (vessel occlusion, AV fistula and pseudoaneurysm) occurred infrequently (15 of 314 patients, 4.8%) and was only noted in patients where the accessed vessel had high grade stenosis and intervention. The most common complication was vessel occlusion (9 of 314 patients, 2.9%), as diagnosed by a follow-up ultrasound. The mechanism involves arterial wall injury via sheath placement/manipulation and angioplasty, thrombus formation and the residual limitations of a mature patent hemostasis method. Note that all patients received therapeutic intravenous heparin and anti-spasmodic medications, as adapted from transradial artery access proceedings.

Embolization events were unexpectedly not encountered, but perhaps this was due to a sheath occupying the single remaining outflow vessel where frequent “sheath bleed back” or aspiration were performed with each catheter or device use or exchange, which may alleviate the collection of debris and thrombus. AV fistula (with 50% noted to have spontaneous resolution) and pseudoaneurysm requiring thrombin injection were observed and were manageable. Despite these post-procedural vascular disruptions, there were no major clinical consequences or limb amputation events at the 6-month follow-up.

In this study, only patients with access to the anterior tibial and peroneal arteries had access vessel complications. However, this may, in part, be due to a minority of patients (10.2%) in this cohort having the posterior tibial artery and dorsalis pedis artery as the remaining non-occluded vessel for access. There was no noticeable difference in relation to access vessel complication, procedural use of atherectomy, the presence of concomitant above- and below-knee PAD, and/or sheath size used. Only 4 and 5 French (outer diameter) sheaths were utilized, and sheath size was chosen at the time of ultrasound guided access and anticipation of needed revascularization equipment.

Retrograde tibial-pedal access has often been considered analogous to the transradial access for coronary angiogram and intervention. Grossly similar rates of tibial vessel complications in our cohort are seen compared with previously published transradial access data. Radial artery occlusion rates have varied from 0.8–10% [20,21,22], pseudoaneurysm occurred in 0.6% of patients and AV fistula occurred in 0.04% of patients [23]. Our transpedal experience parallels the vessel complications reported in the transradial access literature.

Moreover, the concept of safe transulnar catheterization in patients with ipsilateral radial artery occlusion runs analogous to the safe retrograde pedal access in patients with one remaining infra-popliteal runoff vessel, as demonstrated on this study. In the human forearm, the anterior interosseous artery plays an important role in maintaining perfusion to the hand; similarly, intraosseous blood flow within the human tibia, along with collaterals to microcirculation, has been demonstrated to compliment arterial flow to the foot [24,25,26]. Thus, even though there were nine patients with documented access vessel occlusion, there were no limb amputation events, which may be attributed to the compensatory and preserved intraosseous circulation.

Low volumes of intravenous contrast administration were afforded by this access approach. The average amount of total administered contrast was merely 42.2 mL, considering the diagnostic and interventional complexity of this cohort. Contrast “washout” is minimized with this technique compared with traditional or antegrade femoral. All manual controlled contrast injections can be performed through small catheters with 1:1 contrast to saline mix, “dilated contrast”, and this has allowed for excellent diagnostic angiogram images. Close to half of the study’s patients had an estimated glomerular filtration rate of <60 mL/min/1.73 m^2^, and given the strong association of chronic kidney disease and negative clinical outcomes in PAD patients, judicious use of intravenous contrast remains ideal [27].

Our large retrospective study differs from previous reports on the successful application of retrograde pedal access interventions. This study harnessed consecutive patients selected for peripheral angiogram and intervention, and the final inclusion was demonstrated by angiogram-proven anatomy of two vessel infra-popliteal occlusions. To our knowledge, this is the largest dataset of advanced infra-popliteal and multi-level PAD patients who underwent endovascular procedures via this access approach. Our study included patients largely with an RC3 to RC5 range of symptoms; this expands the knowledge on previous reports that have demonstrated successful revascularization with RC 5–6 patients using retrograde pedal access [3,28] and adds support to the adaptation of this access approach to a more broader outpatient demographic with symptomatic PAD.

### Limitations

This study was limited due to its retrospective nature involving a single outpatient center of experienced operators and collaborative skilled nursing with proficient volume and expertise in this access approach. A lack of a comparison arm utilizing other access approaches is a limitation, but the low clinical event rates in this study suggest a strong signal for safety. A prospective and randomized investigation, in particular, against transfemoral access for PAD therapies may be insightful.

Secondly, 12 out of 84 patients with SFA/popliteal CTO required a second vascular access (left radial artery) to successfully cross the occlusion. This illustrates that crossing SFA/popliteal CTO will not be feasible in a minority of patients via a tibial-pedal only access approach; proceduralists adopting routine transpedal access will need to be well versed with antegrade techniques including CART in these instances. Furthermore, this cohort demonstrates an effective non-femoral artery approach (sparing transfemoral artery access complications) via the combination of a transpedal and left radial artery access in these difficult SFA/popliteal CTO cases.

Lastly, our study on the safety endpoints for transpedal access included a clinical and ultrasound follow-up period of 6 months. Although there were no catastrophic complications, including the development of procedural-related wounds or amputations at 6 months, the follow-up duration within this study may not fully capture the deterioration of PAD or amputation events. Nevertheless, this duration should be compared with a much shorter 1-month follow-up from studies highlighting the safety and benefits of the transradial experience, which had successfully challenged a previously default method of transfemoral access for coronary artery disease and intervention [29].

## 5. Conclusions

This study achieves the intention of illustrating the feasible and safe application of routine retrograde transpedal access by demonstrating this technique among outpatients with severe multi-level and infra-popliteal disease involving two runoff vessel chronic total occlusions. This study further substantiates a pedal-first approach for lower extremity endovascular PAD therapies, even in instances with a single remaining vessel infra-popliteal artery runoff.

## Figures and Tables

**Table 1:** Baseline Characteristics (n = 314).

Age	77.9 years (range 46–100 years)
Sex	Male 152 (48.4%)
	Female 162 (51.6%)
Race	Asian 181 (57.6%)
	Black 53 (16.9%)
Smoker	Active 30 (9.5%)
	Former 136 (33.8%)
	Non-Smoker 178 (56.7%)
Hypertension	281 (89%)
CAD	248 (78.9%)
Diabetes Mellitus	167 (53.2%)
Renal Status	Estimated GFR > 60 mL/min/1.73 m^2^ 180 (57.3%)
	Estimated GFR < 60 mL/min/1.73 m^2^ 134 (42.7%)
Rutherford Class	Class 3 88 (28%)
	Class 4 207 (66%)
	Class 4 207 (66%)
	Class 6 1 (0.3%)

**Table 2:** Access Vessel (n = 314).

Anterior Tibial Artery	139 (44.9%)
Peroneal Artery	139 (44.3%)
Posterior Tibial Artery	32 (10.2%)
Dorsalis Pedis	2 (0.64%)

**Table 3:** Access Vessel Complication (n = 15).

Vessel Occlusion	9 (2.9%)
AV fistula	4 (1.3%)
Spontaneous Resolution of AV fistula	2 (50%)
Pseudoaneurysm	2 (0.6%)
Non-cardiovascular death	1 (0.3%)

## Data Availability

The data presented in this study are available on request from the corresponding author. The data are not publicly available due to confidential medical records and are only available with the appropriate legal and ethical permissions.
